# Chlorpromazine activates cGAS-STING signaling and reprograms the immune response in glioblastoma

**DOI:** 10.3389/fimmu.2026.1743232

**Published:** 2026-02-17

**Authors:** Giulia Fanelli, Luisa Gesualdi, Barbara Ascione, Francesca Paolini, Lucrezia Gambardella, Andrea Sacconi, Marco G. Paggi, Paola Matarrese, Claudia Abbruzzese

**Affiliations:** 1 Cellular Networks and Molecular Therapeutic Targets, Istituti di Ricovero e Cura a Carattere Scientifico (IRCCS) - Regina Elena National Cancer Institute, Rome, Italy; 2 Center for Gender−Specific Medicine, Istituto Superiore di Sanità, Rome, Italy; 3 Tumor Immunology and Immunotherapy Unit, IRCCS-Regina Elena National Cancer Institute, Rome, Italy; 4 Biostatistics, Bioinformatics and Clinical Trial Center, Istituti di Ricovero e Cura a Carattere Scientifico (IRCCS) - Regina Elena National Cancer Institute, Rome, Italy

**Keywords:** cGAS-STING pathway, chlorpromazine, glioblastoma, immune checkpoint, immune response

## Abstract

**Background:**

Glioblastoma (GBM), the most common and aggressive primary brain tumor in adults, poses a formidable therapeutic challenge, due to its intrinsic radio- and chemoresistance and its ability to create a hostile, immunosuppressive tumor microenvironment (TME). Drug repurposing has emerged as a promising strategy to fight GBM. In this context, our efforts focused on chlorpromazine (CPZ), a first-generation antipsychotic agent previously shown by us to exert anti-tumor effects in both preclinical and clinical settings.

**Methods:**

We investigated the role of CPZ in remodeling the GBM microenvironment and shaping immune responses using four GBM cell lines, two standard anchorage-dependent models and two patient-derived neurospheres, enriched for tumoral stem cells. We determined cGAS-STING pathway activation and downstream gene expression via flow cytometry and RT-PCR. The cellular secretome following drug treatment was profiled via a luminescence cytokinome assay using a panel of 27 chemokines. Macrophages were phenotyped by flow cytometry using M1 and/or M2 specific markers and, finally, PD-L1 expression was assessed by quantitative flow cytometry and immunoblot analysis.

**Results:**

We demonstrate that CPZ, alone or in combination with temozolomide (TMZ), the current standard of care, activates the cGAS-STING signaling pathway, thus promoting anti-tumor immune responses. Importantly, CPZ counteracts the immunosuppressive effects of TMZ, hindering some TMZ-induced processes as: i) induction of tumorigenic cytokines; ii) macrophage polarization toward a tumor-supportive M2-like phenotype, and iii) increase of PD-L1 expression, a key mechanism of immune evasion.

**Conclusions:**

This study uncovers that CPZ exerts a previously unrecognized anti-cancer immunomodulatory activity, remodeling the immune microenvironment and enhancing the anti-tumor immune response. By overcoming TMZ resistance, CPZ not only exerts a direct anti-neoplastic effect, but also sensitizes GBM cells to standard therapy.

## 1 Introduction

Glioblastoma (GBM) is a devastating global health challenge characterized by aggressive nature and dismal prognosis. Even with the current standard treatment regimen, the Stupp protocol (1), patient outcome remains poor. This underscores the critical need for innovative therapeutic strategies, highlighting the potential of drug repurposing. This strategy leverages existing medications, accelerating the development of safe, effective, and affordable treatments.

In the attempt to repurpose old drugs for GBM treatment, we identified chlorpromazine (CPZ), a well-established medication with a long history of clinical use in treating psychotic disorders, being in use since the 1950s. Its clinical effectiveness arises from the ability to block dopamine D2 receptors in the central nervous system (CNS). Intriguingly, recent research has also suggested a multifaceted anticancer potential for this molecule. Indeed, our *in vitro* studies show that CPZ inhibits GBM cell growth and proliferation, induces cytotoxic autophagy (cell death), subverts energy metabolism, and overcomes temozolomide (TMZ) resistance (2–5). Combining CPZ with TMZ increases DNA damage in GBM cells, weakening, at the same time, their ability to repair such injury (6).

Recent advancements have revealed a deeper knowledge of the link between DNA repair pathways, inflammation and immune responses under stress. Cancer cells frequently develop defects in their DNA damage repair (DDR) machinery, which normally safeguards healthy cells. This failure not only fuels tumor growth but also disrupts the immune system’s ability to respond effectively. While DDR malfunctions are known to promote tumor growth, trigger inflammatory cytokine release, and lead to impaired immune function, DDR deficiency or targeted inhibition can paradoxically, in some cases, directly boost the innate immune system (7).

Cancer cells are constantly bombarded by DNA-damaging insults and both endogenous and exogenous stressors trigger DDR pathways. Aberrant DNA, as well as cytosolic DNA, is captured by the DNA sensor cGAS, thus activating the cGAS-STING signaling axis (8–10). This pathway, in turn, induces the expression of inflammatory genes, leading to two potential outcomes, i.e., cellular senescence or activation of cellular defense mechanisms to eliminate the damaged cell. Activated cGAS-STING signaling network triggers the expression of key genes, including type I interferon, interferon-stimulated genes (ISGs), and inflammatory cytokines, thereby orchestrating an anti-tumor immune response (11, 12). Being cGAS-STING signaling pathway usually inactivated in glial malignancies (13), its activation could result of therapeutic interest.

Tumor microenvironment (TME) plays a critical role in cancer progression. This intricate ecosystem of cells and matrix not only fuels tumor cell proliferation, survival, and migration, but also orchestrates the body’s immune response toward tumors. However, tumor cells can develop mechanisms to evade this immune surveillance, ultimately leading to cancer progression (14, 15). One of the most important immune checkpoints is the Programmed Death 1 (PD-1)/Programmed Death Ligand 1 (PD-L1) pathway. PD-1 acts as a natural brake on T cells, preventing excessive activity in peripheral tissues during infections and autoimmune reactions. However, within the TME, this mechanism is exploited by cancer cells for adaptive immune resistance, as a major immune resistance strategy (16, 17). PD-L1 is a protein expressed by tumor cells (18); in particular, GBM cell lines and biopsies express significant levels of PD-L1, which can also be considered a biomarker directly correlated with tumor grade (19–21).

While immune checkpoint inhibitors (ICIs) have emerged as effective therapeutic approaches for certain solid tumors (such as bladder, kidney, liver, melanoma, and non-small cell lung cancer), monotherapy with anti-PD1/PD-L1 has yielded unsatisfactory results in GBM. This is due to the unique properties of GBM, its high intratumor heterogeneity, the blood-brain barrier permeability, and the characteristics of its tumor microenvironment. Indeed, despite the initial promises, in most trials such treatments failed to significantly shrink tumors or improve patient lifespan (22–24). Nevertheless, several clinical trials are investigating the efficacy of combining ICIs with other treatment types (25, 26).

GBM is considered an immunologically “cold” tumor; indeed, unlike “hot” tumors, GBM exhibits an immune phenotype characterized by a substantial paucity of tumor-infiltrating lymphocytes (TILs), PD-L1 expression on immune cells and genomic instability (27). In fact, GBM is characterized by a complex microenvironment, harboring a diverse cellular population that includes infiltrating immune cells (like macrophages), resident immune cells, vascular cells, and other glial cells. Notably, tumor-associated macrophages (TAMs) represent the most abundant infiltrating immune cell type, making up roughly 30–40% of the cells in the whole GBM mass (28, 29). These TAMs interact with tumor cells favoring their growth and progression (30). There are two subtypes of TAMs: M1 macrophages, with a tumor-suppressive function, and M2 macrophages, with a tumor-supportive role, respectively inhibiting or promoting tumor proliferation, invasion, metastasis and angiogenesis (31, 32). This is the reason why new treatments are being developed to flip the switch on M2 TAMs, turning them into tumor-fighting M1 macrophages.

The present *in vitro* study delves into the intricate interplay between DNA damage signaling pathways and cellular immunity in GBM cells following CPZ treatment, with or without concomitant TMZ administration. This newly discovered cellular mechanism adds a new and significant piece to the puzzle unraveling the CPZ-mediated antitumor activity, so renewing promise for GBM treatment.

## 2 Materials and methods

### 2.1 Cell lines

Anchorage-dependent GBM cell lines, including U-87 MG and U-251 MG, were cultured in Dulbecco’s Modified Eagle’s Medium (DMEM) supplemented with 10% fetal bovine serum (FBS) and a penicillin/streptomycin solution, according to established protocols. Anchorage-independent neurospheres TS#1, TS#163, and TS#83 are patient-derived cell lines that have undergone prior characterization and were cultured as previously detailed (33). All cell lines, when treated, were exposed to a drug dose corresponding to their IC30 (**Supplementary Table S1**).

To generate 3D U-251MG, we first detached cells from the flask via trypsinization. The resulting cellular suspension was centrifuged at 300 RCF for 5 min. Cells were then plated at a density of 2x10^5^ cells in 10 mL of Stem Medium into an Ultra Low T25 culture flask and incubated at 37 °C with 5% CO_2_ for three weeks. During this period, the culture medium and growth factors were systematically added to support the formation of U-251 MG spheroids. The spheroids were subjected to mechanical dissociation about once a week.

### 2.2 Drugs

CPZ, commercially known as Largactil, was acquired from Teofarma S.R.L., Valle Salimbene (PV), Italy. This was provided as a 25 mg/ml solution, equivalent to 78 mM. TMZ (Selleckchem, Houston, TX, USA) was reconstituted in DMSO to create a 150 mM stock solution. In each experiment, GBM cells, as well as macrophages, were treated with CPZ for 48 h and with TMZ for six days; when treated with a combination of both drugs, GBM cells were first exposed to TMZ for 96 h, followed by the addition of CPZ for an additional 48 h, for a total exposure time of 6 days.

### 2.3 PBMC isolation and purification

Peripheral blood mononuclear cells (PBMCs) were isolated from 12 healthy male donors (average age 45.5 ± .13.9) - upon consent in accordance with the principles of the Declaration of Helsinki - by Ficoll-Hypaque gradient (Sigma-Aldrich, St. Louis, MO). Untreated and highly purified CD14+CD16- monocytes were purified by negative immunomagnetic selection using the EasySep Human Monocyte Isolation Kit (STEMCELLTechnologies, Cambridge, UK). CD14+ monocytes were seeded for 7 days in RPMI 1640 medium with 2 mM/L L-glutamine (Life Technologies, Frederick, MD) supplemented with 10% heat-inactivated FBS (Sigma-Aldrich), 100 U/ml penicillin (Life Technologies), and 100 mg/ml streptomycin (Life Technologies) and 50 U/ml GM-CSF (PeproTech, Rocky Hill, NJ) at 37 °C in a humidified 5% CO2 incubator to obtain M0 macrophages. To differentiate M0 into M1 macrophages, we used a combination of GM-CSF (20 ng/ml) (PeproTech), interferon-γ (IFN-γ, 20 ng/ml), interleukin 6 (IL-6, 20 ng/ml), and LPS endotoxin (ThermoFisher, 20 ng/ml); for M2 macrophage differentiation a combination of M-CSF, IL-4, IL-6, and IL-13 at a concentration of 20 ng/ml (PeproTech) was used. Subsequently, M0 macrophages were treated with CPZ and M2 macrophages were treated with TMZ, CPZ or their combination and then phenotyped by flow cytometry using CD86 and HLA-DR as M1-specific markers and CD206 and CD163 as M2-specific markers.

Each single experiment was performed in parallel on monocytes isolated from two different donors.

### 2.4 Quantitative flow cytometry

#### 2.4.1 Determination of intracellular proteins

Control and treated anchorage-dependent cells were washed twice in PBS, harvested by a policeman and collected by centrifugation, while neurospheres were directly collected by centrifugation. Pellets were fixed and permeabilized with 2% paraformaldehyde for 30 min and permeabilized by 0.5% (vol/vol) Triton X-100. After washings in PBS, cells were stained with rabbit anti-cGAS (Cell Signaling Technology, Danvers, MA, 1:100), anti-STING (Cell Signaling Technology, 1:100), anti-phospo-STING (Cell Signaling Technology, 1:100) or anti-phospho-IRF3 (Cell Signaling Technology, 1:100) for 45 min a 4 °C. After washings, cells were labeled with an anti-rabbit AlexaFluor-488. As negative controls, cells were incubated with the sole secondary antibody. After labeling, samples were washed and immediately analyzed on a cytometer.

#### 2.4.2 Determination of plasma membrane proteins

Cell surface expression of PD-L1 was quantified by flow cytometry after staining of living cells for 30 min on ice with a monoclonal phycoerythrin (PE)-conjugated antibody anti-human PD-L1 (Thermo Fisher Scientific, Waltham, MA, 1:100). As negative controls (isotype control), we used mouse IgG-PE (BD Biosciences, San Diego, CA, 1:100). Macrophages were phenotyped using the following antibodies: anti-HLA-DR-FITC (BD Biosciences, 1:100) and anti-CD86-APC (BioLegend, San Diego, CA, 1:100) as M1 markers, or anti-CD206-FITC (BioLegend, 1:100) and anti-CD163-APC (BioLegend, 1:100) as M2 markers.

#### 2.4.3 Phagocytosis assays and determination of nitric oxide intracellular production

Phagocytosis was assayed by adding 5×10^5^ pHrodo red E. coli BioParticles (Invitrogen) to 5×10^4^ human primary macrophages. pHrodo red is a low-background pH sensor dye that shows no signal in neutral conditions and only fluoresces in acidic environments. It enables better discrimination of internalized cargo from outside the cell because it has an approximate pKa of 5 and does not fluoresce until it enters the late endosome and lysosome. Phagocytosis was stopped at 1hour by washing away the excess beads with PBS and adding a PBS solution containing trypsin (1.5 g/L)-EDTA (0.4 g/L). The macrophages were subsequently incubated 5 min at 37 °C with 2 μM of diaminofluorescein-2 diacetate (DAF-2 DA). DAF-2 DA is a fluorescent cell permeable dye that is hydrolyzed to form a cell impermeable molecule which retains inside the cells to detect intracellular nitric oxide (NO). NO production is proportional to the amount of fluorescence emitted in the FL1 channel (like FITC). After labeling, samples were washed and immediately analyzed on a cytometer and NO production was simultaneously assessed in macrophages that had or had not incorporated fluorescent beads. Macrophages that appeared to fluoresce red were considered phagocytic because intracellular particles continued to fluoresce, while extracellular particles became non-fluorescent.

### 2.5 RNA extraction and RT-PCR

Total RNA was isolated from all GBM cells, including untreated controls and those treated with CPZ, TMZ, or their combination, using the miRNeasy Extraction Kit (QIAGEN, Hilden, Germany). The extracted RNA served as a template for retrotranscription, followed by real-time polymerase chain reaction (RT-PCR) to quantify the transcriptional levels of genes downstream of the cGAS-STING axis activation. Quantification of RT-PCR data using the 2^−ΔΔCT^ method yielded fold changes relative to control cells, which are arbitrarily reported as 1.0. *GAPDH* was employed to normalize CT values; all primers used are listed in **Supplementary Table S2**.

### 2.6 Cytokinome assay

GBM cells were plated on 35 mm dishes and then exposed to CPZ, TMZ, a combination of both drugs or vehicle control. After treatment, supernatants were harvested and centrifuged at 300 RCF for 5 min to pellet and remove any remaining cell debris. Simultaneously, anchorage-dependent cells were detached with trypsin/EDTA and counted for downstream normalization, while neurospheres were mechanically dissociated into single cells, resuspended, and quantified.

Supernatants were analyzed using a Bio-Plex Pro Human Cytokine Screening 27-Plex panel (Bio-Rad Laboratories, Hercules, CA) and cytokine/chemokine quantification using standard curve was performed on a Bio-Plex Magpix platform (Bio-Rad), according to the manufacturer’s guidelines.

### 2.7 Immunoblot analysis

Treated and untreated GBM cells were lysed in RIPA buffer in the presence of a cocktail of protease and phosphatase inhibitors. Total protein extracts were separated by gel electrophoresis (SDS-PAGE), transferred onto a PVDF membrane, saturated with a blocking solution (Bio-Rad) for 5 min at room temperature and probed with anti-PD-L1 (Cell Signaling Technology, 1:1000) and anti-GAPDH (Cell Signaling Technology, 1:1000) rabbit monoclonal antibodies. This PD-L1 mAb effectively detects both non-glycosylated (~33 kDa) and glycosylated (~45–50 kDa) forms of endogenous PD-L1 protein in tumor cells. Glycosylation of PD-L1 is crucial for its stability and allows tumor cells to evade immune surveillance by interacting with PD-1.

### 2.8 Knockdown of the cGAS-STING pathway

To investigate whether PD-L1 modulation was dependent on the cGAS-STING axis, we inhibited STING using siRNA-mediated knockdown. GBM cells (U-87 MG and U-251 MG) were seeded in 35 mm dishes (1.2 x 10^5^ cells/dish) and transfected with either STING-targeting siRNA or a non-targeting control (Dharmacon, Lafayette, CO, USA) using Lipofectamine RNAiMAX (Invitrogen, Waltham, MA, USA) according to the manufacturer’s instructions. Twenty-four hours post-transfection, cells were treated with CPZ (48 h), TMZ (6 days), or the TMZ+CPZ combination (4 days TMZ followed by 2 days CPZ). For the extended drug treatments, siRNA transfections were refreshed at the 72-hour mark to ensure sustained gene silencing.

Subsequently, the silenced and pharmacologically treated cells were subjected to flow cytometry and western blot analysis to evaluate PD-L1 expression.

### 2.9 Statistical analysis

Results from FACS, Real-Time PCR, and Western blotting were quantified as the mean ± Standard Deviation (SD) for each experimental group, relative to its corresponding control. To ensure the robustness of our findings, all experiments were independently replicated at least three times. Data were analyzed using an unpaired, two-tailed Student’s t-test (Prism v9, GraphPad Software Inc., San Diego, CA, USA) for pairwise group comparisons. To determine if variables followed a normal distribution Shapiro-Wilk’s test was used. Homoscedasticity was assessed by Bartlett’s test or F-test. For flow cytometry experiments, samples were acquired with a FACScalibur cytometer (BD Biosciences) equipped with a 488 nm Argon laser and with a 635 nm red diode laser and analyzed using CellQuest software (BD Biosciences). At least 20,000 events were acquired for each sample. Statistical significance was set at p < 0.05. In Figures, symbols indicate significance compared to control (asterisks *) or TMZ alone (hash symbols ^#^). p ≤ 0.05 (*;^#^), p ≤ 0.01 (**;^##^), and p ≤ 0.001 (***;^###^).

## 3 Results

### 3.1 CPZ and TMZ activate the cGAS-STING signaling pathway in GBM cells

**Figure 1:**
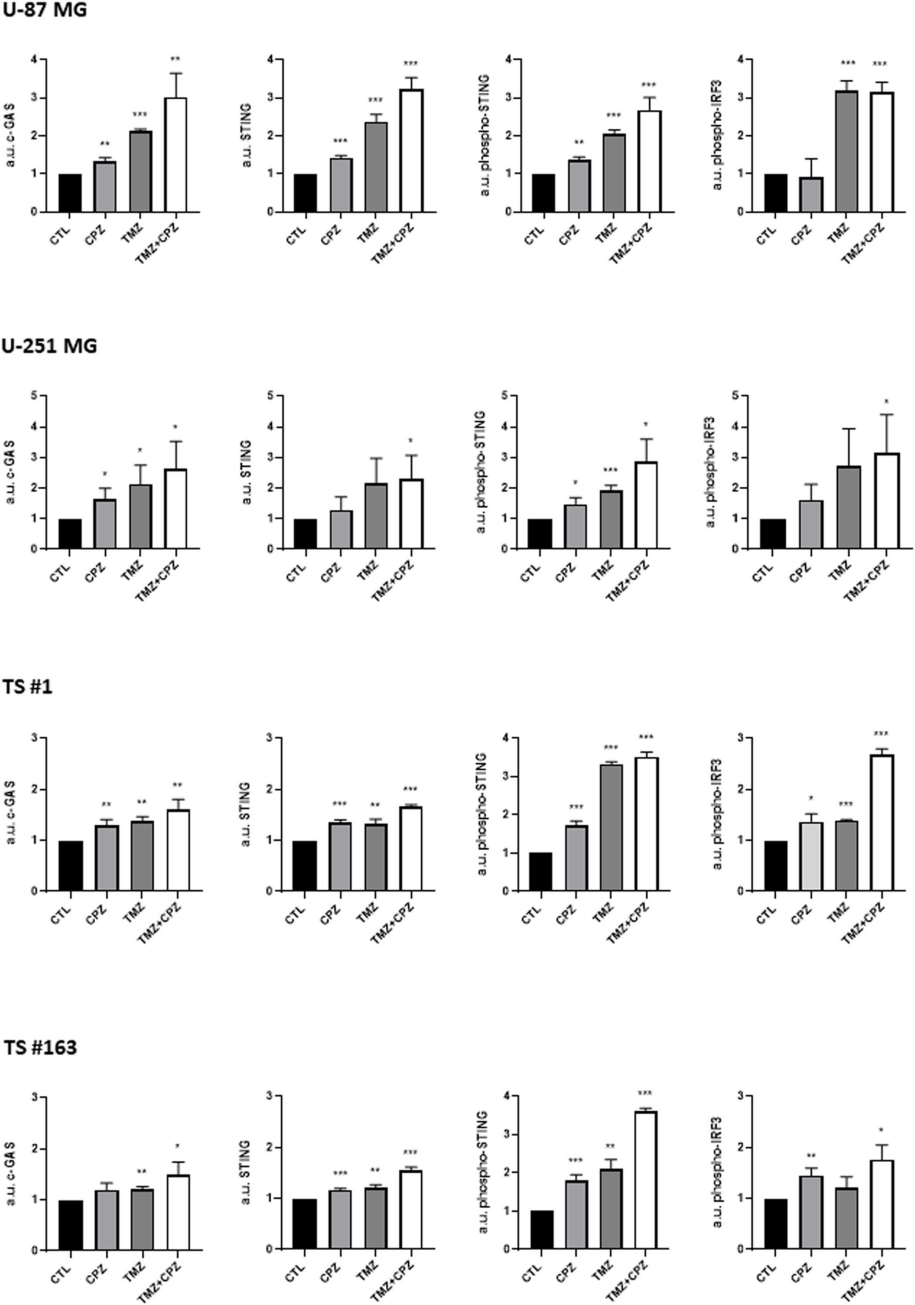
CPZ and TMZ activate the cGAS-STING signaling pathway in GBM cells. To assess the expression of key markers of the cGAS-STING pathway, all GBM cells, both treated and untreated, were subjected to FACS analysis. For drug treatments, GBM cells were exposed to their IC30 dose of CPZ for 48 h or TMZ for 6 days. For the combination treatment, GBM cells were first exposed to TMZ for 96 h, followed by the addition of CPZ for additional 48 h, resulting in a total exposure time of 6 days. To accurately capture phospho-STING expression in neurospheres, we had to reduce the drug exposure times. A preliminary time-course experiment revealed an early, transient activation of phospho-STING occurring before the later increase in phospho-IRF3. Based on this, we shortened the exposure to 3 days for TMZ and 16 h for CPZ. Bar graphs were generated by pooling data from three independent experiments and are presented as the mean ± SD of the median fluorescence intensity. Statistical significance was determined using t-test, and asterisks indicate significance levels, compared to each relative untreated control, as follows: *p < 0.05, **p < 0.01, and ***p < 0.001.

Given the established role of DDR defects in promoting tumor immunogenicity via cGAS-STING pathway activation, we assessed the expression of three key pathway components, i.e., cGAS, total and phosphorylated STING, and phospho-IRF3, by a cytofluorimetric assay in GBM cells treated with CPZ, TMZ, or their combination (**Figure 1**; **Supplementary Figures S1A-D**). In the anchorage-dependent U-87 MG and U-251 MG GBM cell lines, all analyzed factors were upregulated. In this setup, TMZ, consistent with its alkylating nature, induced a stronger pathway activation than CPZ.

Similarly, patient-derived GBM spheroid cell lines TS #1 and TS #163 exhibited increased levels of cGAS, total and phospho-STING, and phospho-IRF3. Interestingly, neurospheres displayed an early transient activation of phospho-STING after 16 h of CPZ treatment and 3 days of TMZ treatment, potentially reflecting a more rapid pathway induction within this 3D model configuration.

Altogether, these data highlighted the ability of both CPZ and TMZ to induce the cGAS-STING signaling pathway in GBM cells with the combination treatment exerting a markedly stronger effect. This suggests that both drugs can act as intrinsic triggers of innate immunity, thereby potentiating GBM cells immunogenicity.

### 3.2 Activated cGAS-STING axis promotes anti-tumor immune response in GBM cells

**Figure 2:**
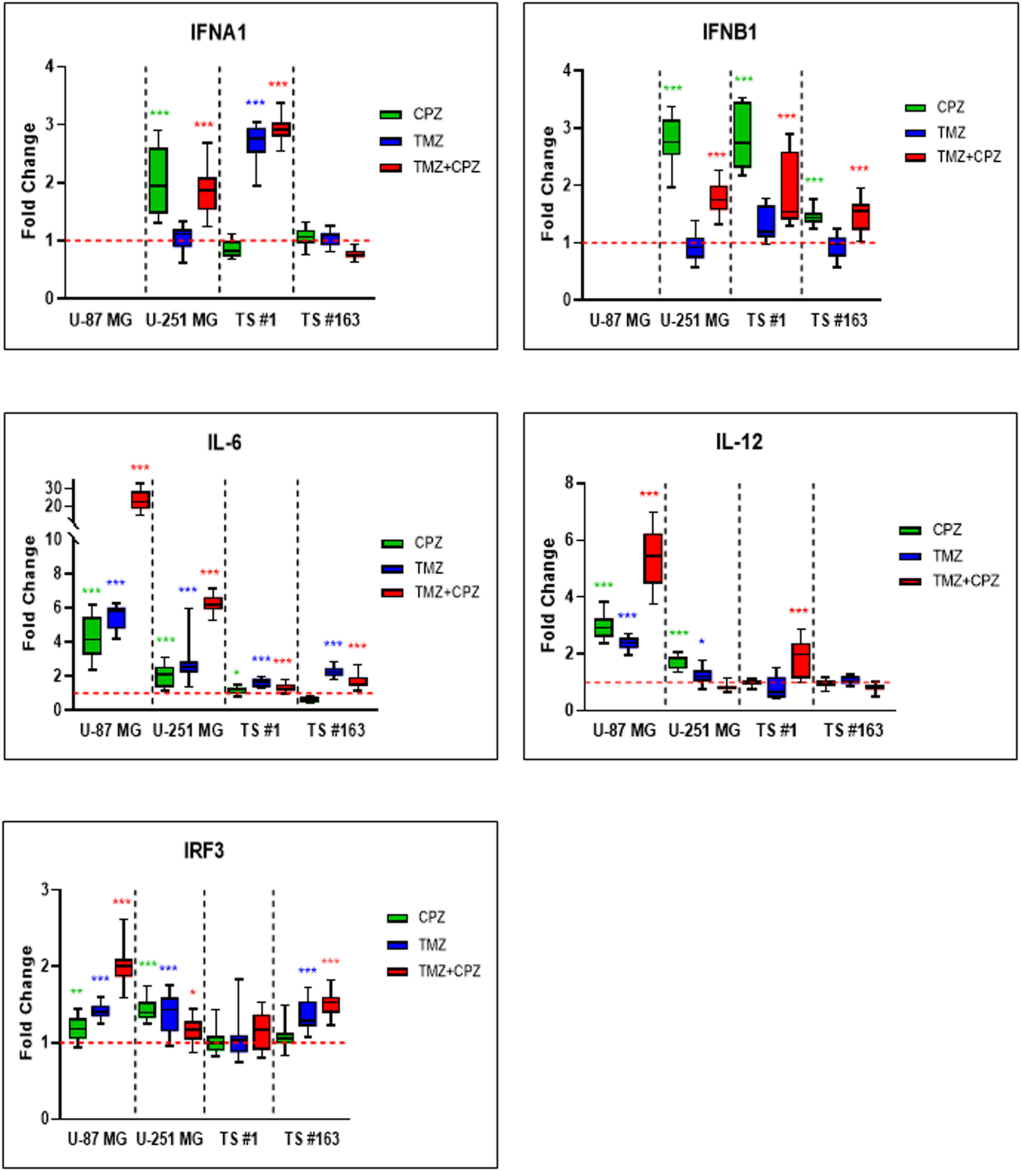
Activated cGAS-STING axis promotes anti-tumor immune response in GBM cells. RT-PCR analysis of key anti-tumor immunity genes, including *IRF3, IFN-α, IFN-β, IL-6, and IL-12*, in GBM cells revealed an overall trend of increased expression, following all treatments. Cells were treated with TMZ IC30 for 6 days, CPZ IC30 for 48 h or with IC30 TMZ+CPZ combo for 96 h+48 h, respectively. Fold changes from three independent experiments are shown in box plots, with the untreated control normalized to 1.0. The box represents the median, and whiskers extend from the minimum to the maximum value. Statistical significance compared to untreated control was assessed using an unpaired Student’s t-test. Asterisks indicate the p-value thresholds vs the untreated control: *p < 0.05; **p < 0.01; ***p < 0.001.

Through its canonical pathway, STING recruits IRF3 to the signalosome, leading to IRF3 phosphorylation, dimerization, and nuclear translocation. This process drives the induction of type I *IFN*s and various *ISG*s, a process that occurs in parallel with NF-κB activation and subsequent proinflammatory cytokine production (9). Therefore, we examined, through RT-PCR, the transcript levels of crucial genes involved in anti-tumor immunity, namely *IRF3*, type-I Interferons (*IFNA1* and *IFNB1*), and cytokines like Interleukins (*IL-6* and *IL-12*). As shown in **Figure 2**, drug treatments with CPZ, TMZ, and combined therapy consistently increased transcriptional expression of these genes in GBM cells. Notably, type I interferons mRNAs were undetectable in the U-87 MG cell line. Furthermore, the increase in interleukin mRNA levels was more pronounced in anchorage-dependent GBM cell lines than in patient-derived neurospheres.

### 3.3 Drug-mediated alterations of the GBM cells cytokinome

**Figure 3:**
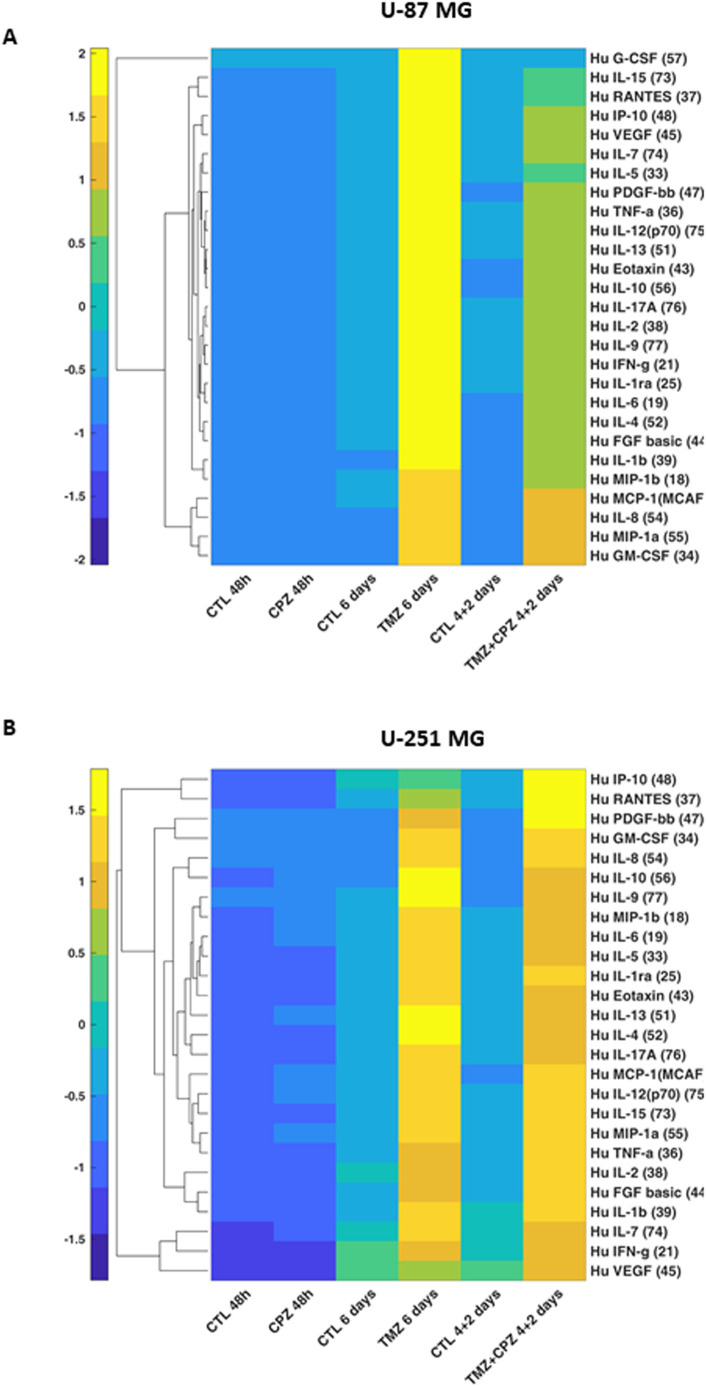
Drug-mediated alterations of GBM cells cytokinome. Supervised hierarchical clustering of 27 cytokine levels in conditioned media from anchorage-dependent GBM cells (U-87 MG in A and U-251 MG in B). Cells were treated with CPZ for 48 h, TMZ for 6 days, or a combination of TMZ and CPZ (4 + 2 days). Cytokine luminescence intensity was standardized, normalized to cell counts, and clustered using Euclidean distance. Numbers in brackets for each cytokine indicate the bead-region identifier assigned to that analyte by the Bio-Plex Cytokine kit for MAGPIX acquisition software. The heatmap reveals that, while TMZ causes an important increase in soluble factors, CPZ does not substantially alter the cytokinome of GBM cells.

To investigate the impact of CPZ and/or TMZ treatments on TME, we quantified 27 cytokines, chemokines, and growth factors in conditioned media from anchorage-dependent GBM cells and patient-derived neurospheres. Given the varying drug doses and treatment durations, all the data were normalized to cell numbers before generating line-specific heatmaps (**Figure 3**, **Supplementary Figure S2**). The analyzed panel included: a) inflammatory cytokines, especially interleukins; b) chemotactic chemokines, which promote invasion and metastasis, partly via extracellular matrix degradation; and c) pro-invasive and angiogenic growth factors such as VEGF. Collectively, these factors are implicated in malignant progression (**Supplementary Table S3**).

In anchorage-dependent GBM cell lines, CPZ treatment alone induced no substantial changes in the secretome profile. Conversely, TMZ, alone or in combination with CPZ, markedly increased the release of several analytes, consistent with a therapy-evoked inflammatory/immunosuppressive secretome associated with treatment resistance and disease progression (34) (**Figures 3A, B**). Remarkably, in the U-87 MG GBM cell line, CPZ effectively counteracted the TMZ-induced effect when administered together (**Figure 3A**).

In GBM neurospheres, the overall direction of change mirrored that of anchorage-dependent cells but with reduced magnitude and less pronounced drug-specific differences (**Supplementary Figures S2A, B**). This is consistent with the stem-like phenotype and intrinsic chemoresistance characteristic of 3D/patient-derived GBM models (35, 36). For a direct 2D/3D comparison of the same GBM cell line, we analyzed the secretome profile of U-251 MG cells grown as spheroids. Similar to patient-derived neurospheres, U-251 MG-derived spheroids exhibited a comparable yet blunted secretory profile (**Supplementary Figure S2C**). To corroborate the secretome profile in GBM neurospheres, we used an additional patient-derived GBM cell line, TS #83 (**Supplementary Figure S2D**). Interestingly, its cytokinome pattern closely resembled that of anchorage-dependent GBM cell lines, specifically the U-87 MG profile, which further highlighted the marked variability among these GBM patient-derived neurospheres.

Finally, untreated neurospheres displayed a distinct basal cytokinome profile, characterized by a reduced number of secreted cytokines, a feature most evident in the TS #1 cells (**Supplementary Figure S2**).

With the aim of complementing the heatmap data, **Supplementary Table S3** provides individual tables for each GBM cell line, detailing quantitative fold-changes for the expressed cytokines.

### 3.4 CPZ opposes macrophage differentiation toward the M2-like phenotype induced by TMZ: phenotypic and functional analyses

**Figure 4:**
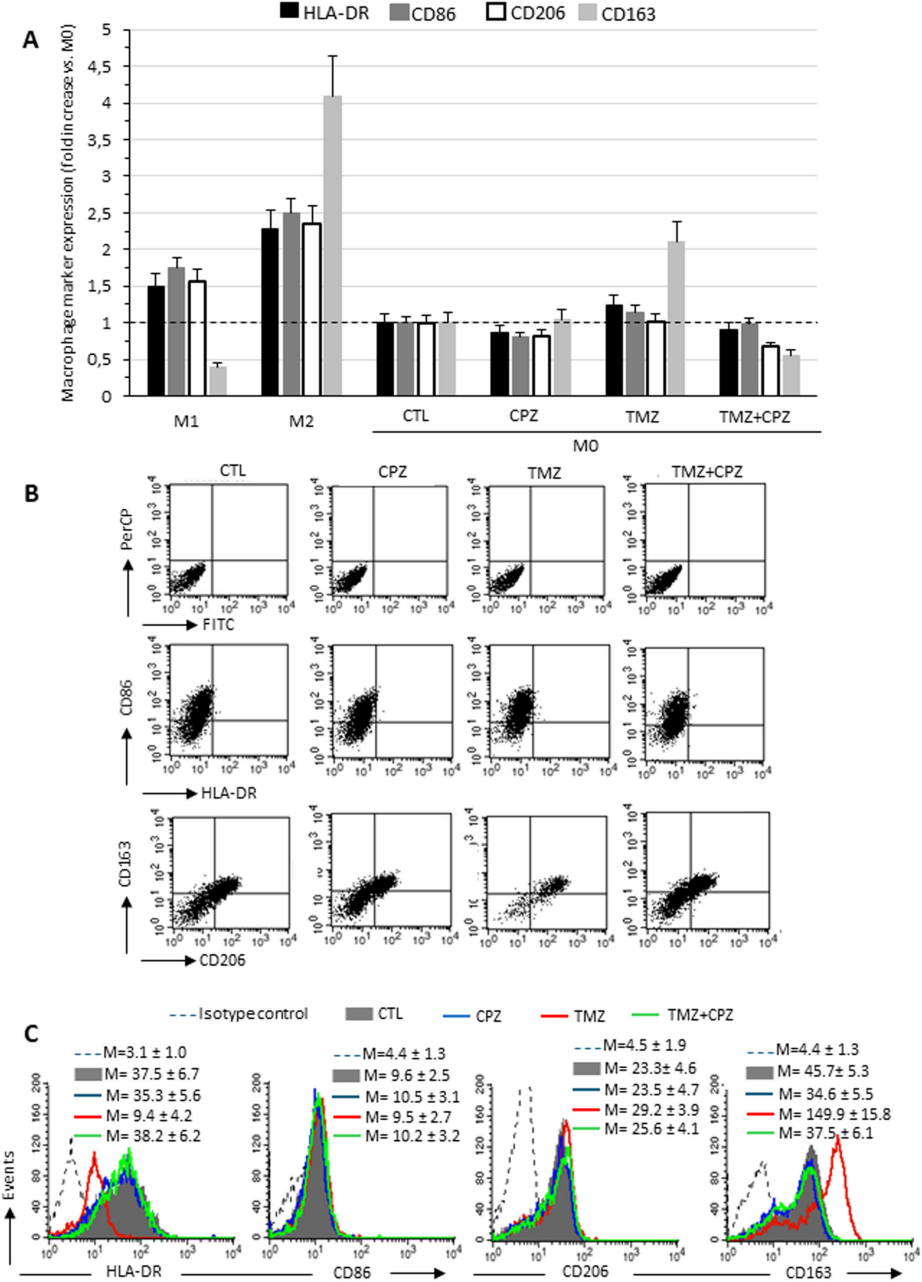
CPZ opposes macrophage differentiation toward the M2-like phenotype induced by TMZ: analysis of phenotypic markers. Flow cytofluorimetry analyses of plasma membrane expression levels of HLA-DR, CD86, CD206 and CD163 in living macrophages. **(A)** The expression levels of the markers are reported in relation to M0 macrophages, in which the median fluorescence intensity values obtained were set equal to 1. Data are the mean ± SD of the results obtained in three experiments. **(B)** Dot plots from a representative experiment following double labeling of M2 macrophages untreated (first column), treated with CPZ (second column), TMZ (third column), or a combination of both (fourth column). The first row shows isotype control, the second row shows cells double labeled with anti-HLA-DR and anti-CD86, and the third row shows cells labeled with anti-CD206 and anti-CD163, as indicated. In the upper right quadrant, double positive HLA-DR/CD86 or CD206/CD163 cells are enclosed. **(C)** The histograms showing the expression of the four markers separately in the different experimental conditions, as specified in the legend. The numbers represent the average of the median fluorescence intensity values obtained in three independent experiments ± SD.

It has been previously reported that TMZ treatment promotes M2-like polarization of infiltrating macrophages (37), which in turn contributes to TMZ resistance, thus establishing a malicious loop (38). Furthermore, elevated levels of TGF-β1 have been positively correlated with tumor growth, invasiveness, and therapeutic resistance in GBM (39). Based on these findings, we investigated whether CPZ could counteract the M2 macrophage phenotype. To this end, we treated M2 macrophages with CPZ and assessed the possible phenotypic switch towards the M1 state. Polarization was assessed by comparing the expression on the plasma membrane of the M1 markers HLA-DR and CD86, and of the M2 polarization markers, CD206 and CD163. Semiquantitative flow cytometry analysis revealed no significant alterations in plasma membrane HLA-DR, CD86, and CD206 levels, while a clear and statistically significant reduction of the M2 marker CD163 was observed (**Supplementary Figures S3A-C**). Because macrophage polarization is not a binary process, and macrophages may co-express M1 and M2 markers (40), to better understand the effects of CPZ, alone and in combination with TMZ, we adopted a different experimental strategy. For this, M0 macrophages were exposed to pharmacological treatments to observe the subsequent differentiation toward M1- or M2-like phenotypes (**Figure 4**). **Figure 4A** shows the expression of the above M1 (HLA-DR and CD86) and M2 (CD206 and CD163) markers in M0 macrophages treated with CPZ, TMZ, or their combination in comparison with untreated M0 (CTL), and with M1 and M2 macrophages obtained from three different healthy donors. First of all, a consistent increase in all analyzed markers was observed in both M1 and M2 phenotypes compared to M0 macrophages (**Figure 4A**, **Supplementary Figure S4**), except for the M2 marker CD163, whose expression was significantly reduced in M1 macrophages. The described increase was significantly higher in M2 macrophages, particularly for CD163 (**Figure 4A**). Compared to untreated M0 macrophages, CPZ alone did not induce appreciable changes, while TMZ significantly increased the membrane expression of the M2 marker CD163, which was effectively counteracted by CPZ (**Figures 4A, B**).

Taken together and compared with data from reference samples (M0, M1 and M2 macrophages), our results suggest that CPZ did not promote differentiation when administered to quiescent macrophages (M0), while effectively counteracted the TMZ-driven switch towards the M2-like phenotype.

**Figure 5:**
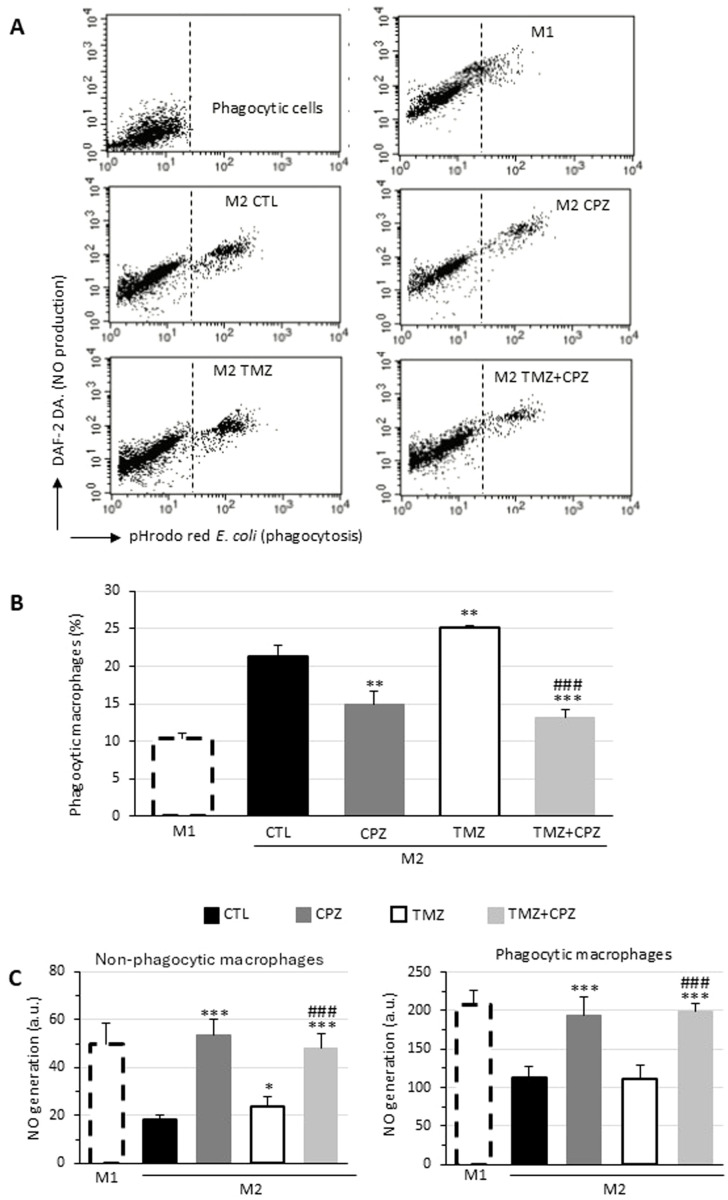
CPZ opposes macrophage polarization toward the M2-like phenotype induced by TMZ: functional analyses. Phagocytosis of *E*. *coli* pHrodo Red particles by macrophages. M1 or M2 macrophages incubated with *E*. *coli* particles for 1h and stained with DAF-DA2 to reveal NO production were analyzed by flow cytometry. Representative dot plots showing the red fluorescence emission due to pHrodo Red internalization (abscissa) and the green fluorescence emitted by DAF-DA2 proportional to the amount of NO generated by the cells (ordinate). The top left panel represents unstained cells (negative control). **(B)** Bar graph showing the percentage of cells that internalized *E*. *coli* particles in different experimental conditions. **(C)** Bar graph reporting NO production in non-phagocytic (left panel) and phagocytic (right panel) macrophages expressed as median fluorescence intensity. Results were obtained in three independent measurements and expressed as mean ± SD. Statistical significance was determined using t-test. In **(B, C)** asterisks indicate the p-value thresholds vs the untreated control and hash symbols display the same p-values in combo treatment vs TMZ alone. p ≤ 0.05 (*;^#^), p ≤ 0.01 (**;^##^), and p ≤ 0.001 (***;^###^).

Since M1 and M2 macrophages, in addition to expressing distinctive markers, represent two different functional types, we analyzed the effects of treatment with CPZ, TMZ, or their combination on phagocytosis and NO production (**Figure 5**). Consistent with literature data (41), we observed that M1 macrophages showed a lower phagocytic activity, but greater NO production compared to both untreated M2-like macrophages (CTLs) and TMZ-treated macrophages. CPZ administration, either alone or in combination with TMZ, resulted in a reduction in phagocytosis and an increase in NO production to levels not dissimilar to those observed in M1-like macrophages. Interestingly, in all experimental conditions, NO production was greater in the fraction of phagocytic cells compared to non-phagocytic cells within the same sample (**Figures 5A, C**). These functional data appear to confirm what had already been observed at the phenotypic level.

### 3.5 CPZ reduces PD-L1 expression in GBM cells

PD-L1 is considered the most critical immune checkpoint molecule. Its interaction with PD-1, a key component of the canonical pathway, effectively suppresses the body’s antitumor immune response (42). In addition, it has been reported that membranous PD-L1 is increased in TMZ-treated GBM cells (43).

**Figure 6:**
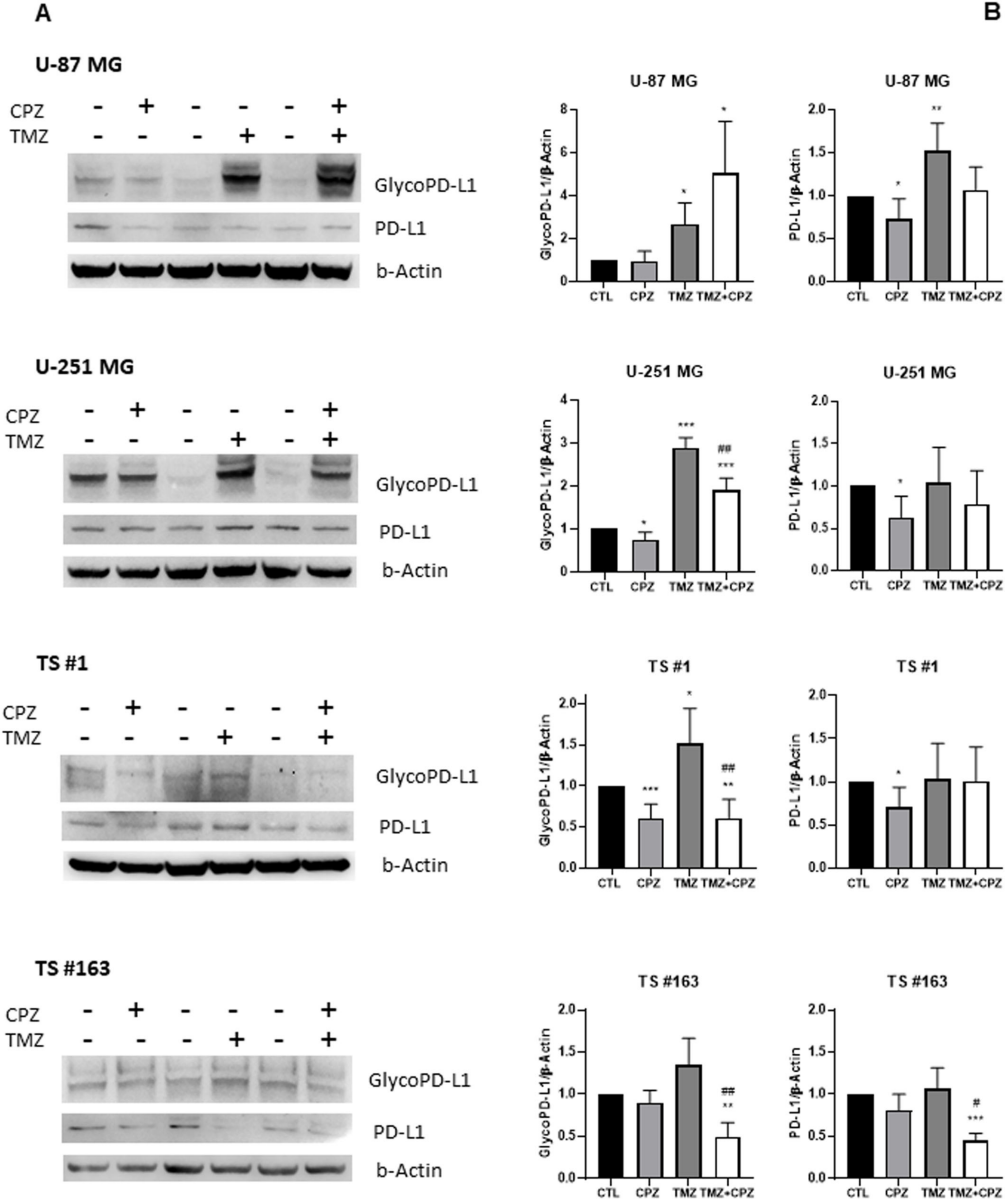
Drugs induced modifications of PD-L1 expression. The protein levels of PD-L1 were assessed by western blotting in all GBM cell lines following drug treatment (CPZ for 48 h, TMZ for 6 days, TMZ+CPZ for 4 + 2 days with IC30 doses). β-actin served as the loading control for relative quantification. **(A)** Representative western blots show both total and glycosylated isoforms of PD-L1. **(B)** Histograms quantifying by western blotting the expression levels of PD-L1 (glycosylated or not) in treated or untreated GBM cells (t test *p < 0.05; **p < 0.01; ***p < 0.001). Hash symbols represent significant p-values in combo treatment vs TMZ alone (t test ^#^p<0.05; ^##^p<0.01) Bars indicate the mean ± SD, assessed by three independent experiments.

To investigate whether CPZ could counteract the typical immune resistance of GBM cells, we analyzed PD-L1 protein levels in treated tumor cells using Western blotting (**Figure 6**). The antibody used can distinguish between two PD-L1 isoforms on denaturing gel electrophoresis: a lower apparent molecular mass band, indicating the total protein, and higher molecular mass bands, representing the glycosylated endogenous forms. This post-translational glycosylation is critical for maintaining PD-L1’s structural stability and its binding affinity for the cognate receptor, PD-1, which facilitates tumor immune evasion (44).

In line with previous studies (43), we observed an evident increase in the expression of both total and glycosylated PD-L1 isoforms across all GBM cell lines following TMZ treatment compared to the untreated control. Conversely, CPZ treatment led to a reduction across most GBM cells, except for the TS #163 neurospheres, which showed only a slight, non-significant, decrease in PD-L1. Simultaneous exposure of tumor cells to TMZ and CPZ produced cell-type-dependent effects. In anchorage-dependent GBM cell lines, we observed a significant increase in glyco-PD-L1 expression, likely due to the substantial modulation caused by TMZ alone. Importantly, in patient-derived neurospheres, the combination treatment led to a decrease in PD-L1 protein levels.

When comparing TMZ monotherapy to the combination treatment, where CPZ was added during the final 48 hours of exposure, we observed a significant reduction in glycosylated PD-L1 levels across most models, with the exception of the U-87 MG cell line (**Figure 6B**).

All these findings highlight CPZ’s ability to mitigate TMZ-induced mechanisms of immune evasion.

### 3.6 CPZ counteracts TMZ-induced upregulation of PD-L1 on plasma membrane in anchorage-dependent GBM cells

**Figure 7:**
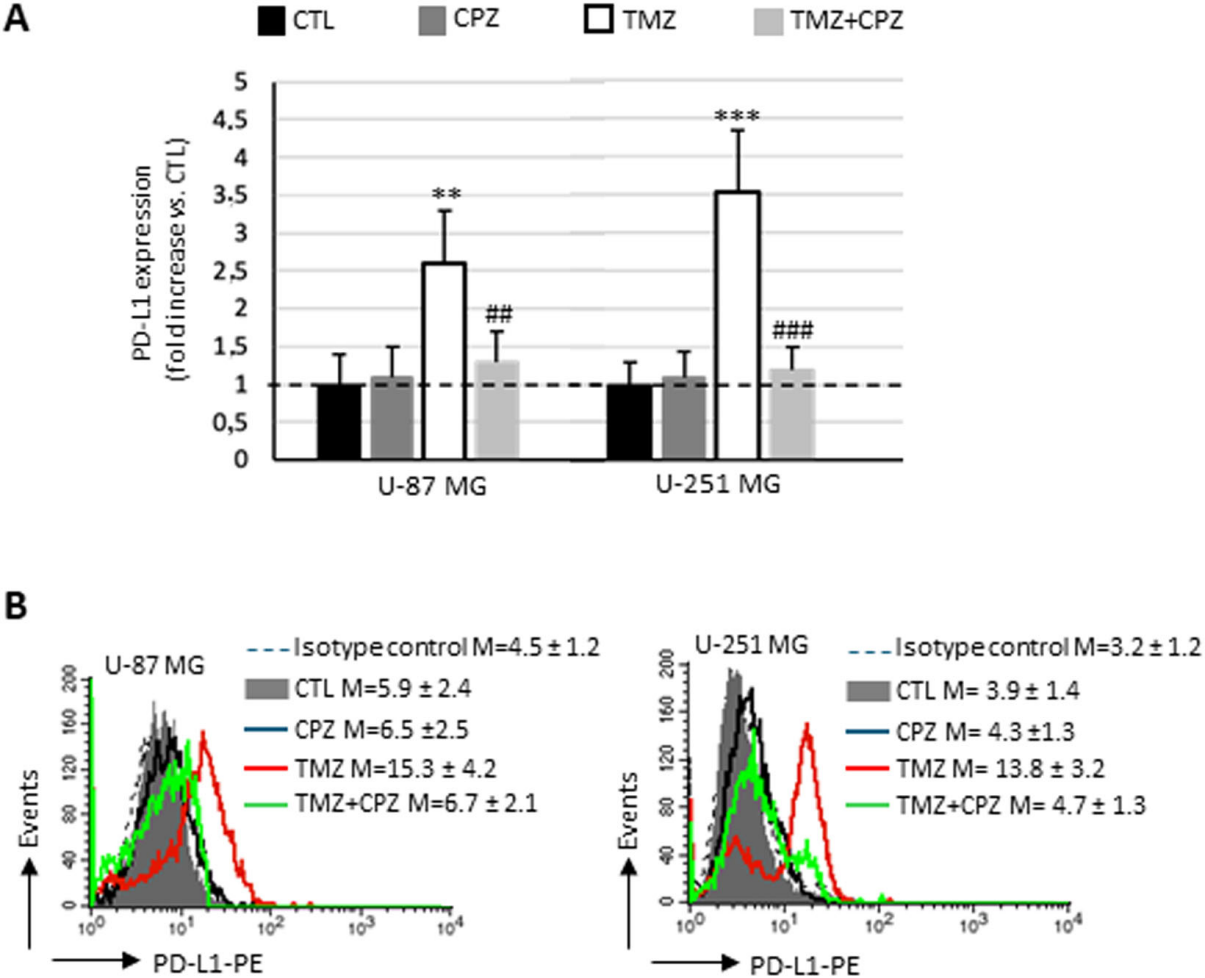
CPZ opposes TMZ-induced upregulation of PD-L1 on plasma membrane in anchorage-dependent GBM cells. **(A)** Bar graph showing the results obtained by a biparametric flow cytofluorimetry analysis of plasma membrane expression of PD-L1 in U-87 MG (left bar panel) and U-251 MG (right panel) untreated or treated with doses corresponding to IC30 of CPZ (48h), TMZ (6 days) or both (4 + 2 days). The expression levels of PD-L1 are reported in relation to untreated cells (CTL), in which the median fluorescence intensity values obtained were set equal to 1. Differences in relative expression were determined by unpaired Student’s t-test and significance levels are denoted as asterisks or hash symbols for comparisons with untreated control or TMZ alone, respectively. p ≤ 0.05 (*;^#^), p ≤ 0.01 (**;^##^), and p ≤ 0.001 (***;^###^). **(B)** The histograms show the expression of PD-L1, separately, in different experimental sets. The numbers represent the average ± SD of the median fluorescence intensity from three independent experiments.

The expression of PD-L1 is found higher on the plasma membrane of TMZ-resistant strains, the anchorage-dependent U87/TR and U251/TR GBM cells (45). Considering this, we performed a semiquantitative analysis by flow cytometry on living cells to investigate the ability of CPZ to counteract the effect of a treatment with TMZ (**Figures 7A, B**). Both untreated U-87 MG and U-251 MG cells expressed negligible amounts of PD-L1 on the plasma membrane (solid gray histograms, compared with the negative control represented by the dashed curve). While 48 h of CPZ treatment failed to induce any significant changes (black lines), in contrast, TMZ prompted a dramatic increase of PD-L1 in both cell lines. Co-administration of CPZ (green curves) was able to completely prevent the TMZ-induced PD-L1expression (red curves) in the plasma membrane of either U-87 MG or U-251 MG, as shown in **Figure 7B**, left and right panel, respectively.

### 3.7 Effect of STING knockdown on PD-L1 expression

To confirm that the observed immunomodulatory effects were strictly dependent on the cGAS-STING axis, we silenced STING expression in anchorage-dependent GBM cells (U-87 MG and U-251 MG) and quantified PD-L1 protein levels via flow cytometry. As shown in **Supplementary Figure S5A**, STING knockdown rescued PD-L1 expression following pharmacological treatment and, once again, the combination treatment further underscored the potency of this effect. We previously demonstrated that the combination of TMZ and CPZ significantly increased STING expression, thereby activating the pathway and attenuating TMZ-induced immune evasion by reducing PD-L1 expression compared to TMZ monotherapy (**Figures 6**, **7**). However, upon STING inhibition, the synergistic effect of CPZ was lost. Under these conditions, PD-L1 expression in the combination group increased when compared with the TMZ monotherapy group, demonstrating that the ability of CPZ to counteract TMZ-induced PD-L1 upregulation was a STING-dependent mechanism. These flow cytometry findings were further confirmed by Western blot analysis in U-87 MG cells subjected to STING gene silencing. As shown in **Supplementary Figure S5B**, protein expression of glycosylated PD-L1 revealed a rescue of the immune checkpoint, particularly evident in the TMZ+CPZ treatment group.

## 4 Discussion

Predicting the clinical efficacy of an immunotherapeutic approach for a cancer patient hinges on understanding the tumor’s “hot” or “cold”, state, which is determined by the intricate interaction of several factors, i.e., the cancer cell’s intrinsic properties, the tumor’s immune profile, its microenvironment, and the underlying signaling pathways (46, 47).

GBM, the most prevalent and malignant primary CNS tumor in adults, poses a major therapeutic hurdle. This is primarily attributed to its significant inter- and intra-tumor heterogeneity and a complex, highly immunosuppressive TME that collectively drives rapid proliferation, invasion, and migration, thus fueling tumor cell survival and growth. Paradoxically, while TMZ remains the standard chemotherapeutic for GBM, it also possesses immunomodulatory properties that may suppress the host immune response and drive a more aggressive tumor phenotype (48). Consistently, recent findings indicate that TMZ facilitates immune escape of GBM cells through PD-L1 upregulation (43). This suggests that a combination therapy of TMZ and an anti-PD-1 antibody may represent a promising strategy for GBM patients (49, 50).

Our previous research on drug repurposing in GBM investigated CPZ, a first-generation antipsychotic used since the 1950s. We rigorously evaluated its antitumoral efficacy *in vitro* in both 2D and 3D GBM cell cultures and simultaneously analyzed its multifaceted molecular mechanisms of action (3–6, 51). Our translational research activity on CPZ culminated in a Phase II clinical trial, a proof-of-concept experimentation which reported a favorable progression-free survival in GBM patients receiving CPZ as an add-on to standard adjuvant therapeutic schedule with TMZ (52).

The present study explores a novel mode of action for CPZ, revealing its critical role in remodeling the GBM microenvironment and shaping immune responses, thereby highlighting a potential new contribution to antitumor efficacy. Indeed, by inducing DDR defects, CPZ triggers cGAS-STING pathway activation (**Figure 1**), which in turn fosters an anti-tumor immune response against GBM cells.

Recognizing the critical role of chemokines in regulating immune cell activation, recruitment, phenotype, and function within the TME, as well as their substantial impact on the efficacy of pro- and anti-tumorigenic immune responses (53), we proceeded to analyze the secretome of GBM cells after drug treatment (**Figure 3**). While TMZ significantly elevated all secreted factors, CPZ did not substantially alter the secretome profile of GBM anchorage-dependent cells. As expected, patient-derived neurospheres yielded a similar cytokinome profile, though results were attenuated by the enrichment of cancer stem cells (CSCs). This is because CSCs, while crucial for tumor development and progression, are not independent entities. Instead, these cells operate within an intricate ecological system, actively remodeling their microenvironment and deriving essential maintenance signals from their niches (54, 55). Collectively, cytokinome analysis suggests that CPZ may impact the TME by reducing the pro-invasive and inflammatory signals induced by TMZ.

TAMs are a major component of the GBM TME and correlate directly to tumor grade and inversely with patient survival (56). GBM cells actively manipulate TAMs to fuel tumor growth by secreting factors that recruit them and cause them to switch toward a pro-tumor M2-like phenotype. These M2-like TAMs, in turn, promote various processes that support cancer progression, such as stemness, proliferation, angiogenesis, cancer cell migration, and immune suppression (32). Hence, targeting TAMs therapeutically is a promising strategy. Potential approaches include: 1) blocking TAM recruitment; 2) reprogramming TAMs, to shift them from tumor-promoting M2-like state to a tumor-suppressing M1-like state; and 3) eliminating M2-like TAMs (31, 32).

Our cytokinome analysis showed that CPZ plays an important part in the TME. Based on this finding, we investigated how CPZ treatment affects macrophages, exploring its potential to reprogram the TAM phenotype. Indeed, following exposure of M2 macrophages to CPZ, we observed a significant reduction in the membrane expression of CD163, a typical M2 marker. When administered to M0 macrophages, CPZ did not induce significant alterations in the analyzed markers, but very effectively opposed the M2-like phenotypic switch induced by TMZ. This suggests a role for CPZ in counteracting the differentiation of macrophages towards the M2-like phenotype, known for their role in supporting tumor growth and drug resistance (**Figure 4**). This interpretation is further supported by the increased levels of interleukin transcripts, as evidenced by RT-PCR analysis (**Figure 2**). Indeed, while IL-6 is a multifunctional, both pro- and anti-inflammatory soluble mediator, IL-12 undoubtedly has a key role in cell-mediated immune response. IL-12, both *in vitro* and *in vivo*, rapidly curbs the activity of tumor-supportive macrophages by decreasing the production of key factors, such as IL-10, MCP-1, migration inhibitory factor, and TGF-β (57). Simultaneously, IL-12 boosts pro-inflammatory and pro-immunogenic responses and triggers potent anti-angiogenic mechanisms (58). Moreover, IL-12 promotes the functional conversion of macrophages from a tumor-supportive to a tumor-suppressive phenotype, a switch subsequently contributing to an antitumor response (57, 59).

To validate the effect of CPZ on immune evasion and drug resistance in tumor cells, we finally analyzed the protein expression of the immune checkpoint PD-L1 in drug-treated GBM cells. This is a critical factor, as PD-L1 autoregulation may create an immunosuppressive microenvironment and promote proliferation, migration, and invasion of GBM cells via the GP130/JAK2/STAT3/IRAK2/IL6 signaling pathway (59). Although the results obtained were not perfectly congruent between Western blot (**Figure 6**) and flow cytometry (**Figure 7**), several technical factors likely account for these discrepancies. Western blot is a semi-quantitative technique designed to assess the presence of a protein in the whole cell lysate, while flow cytometry, at least under our condition, revealed -, always semi-quantitatively -, the amount of protein fraction localized to the plasma membrane. Furthermore, the antibodies used in the two experimental sets recognize different epitopes: the one used for cytometry recognizes an extracellular domain, while that one used for western blot detection targets an intracellular epitope. As a matter of fact, while Western blot provides a population-averaged signal, live-cell flow cytometry offers superior sensitivity and the advantage of single-cell resolution, allowing for the detection of subtle shifts in expression that might otherwise be masked. All these differences, compounded by potential variations in antibody affinity and the inherent sensitivities of each technique, may influence the observed results. However, we would like to emphasize that both techniques highlighted a significant increase in PD-L1 following TMZ treatment, where the most notable result was the significant decrease in active PD-L1 when cells were treated with a combination of CPZ and TMZ.

These results suggest that CPZ may not only contribute to the anti-tumor immune response but also counteract the immune-evasive mechanisms induced by TMZ.

We previously showed that CPZ targets PKM2, stabilizing it in its active tetrameric form (4). This finding supports a new function of CPZ in the immune system. Indeed, previous studies have reported that PKM2 stabilization as a tetramer, or silencing its expression, prevents the upregulation of PD-L1, thus impairing the ability of cancer cells to evade immune surveillance (60, 61). This appears consistent with the molecular structure of CPZ, which is similar to tricyclic H1R1 histamine receptor inhibitors. These molecules are known to inhibit histamine binding to the H1R1 receptor on macrophages, thus hindering the development of a M2-like immunosuppressive phenotype (62).

Despite the clinical relevance of our findings regarding CPZ’s TME-modulating effects, we recognize several methodological limitations. Primarily, the absence of *in vivo* validation and functional T-cell/adaptive immune assays tempers the assessment of the comprehensive immune response However, CPZ’s anti-tumor efficacy has already been demonstrated in an independent clinical trial (52), and our primary focus was on the modifications induced in the TME by treating GBM cells, not the TME’s intrinsic cellular function. Our reliance on a limited number of neurosphere models restricts the biological variation captured, potentially skewing the representation of diverse patient responses or tumor subtypes, and our short-term focus on macrophage effects limits our understanding of durable TME changes. Notably, we observed divergent profiles between anchorage-dependent GBM cell lines and patient-derived neurospheres. This discrepancy is largely driven by the differential regulation of stemness and the simulation of the native TME afforded by the 3D models. Indeed, in 3D cultures, cells aggregate in a spatial organization mimicking the solid tumor structure that facilitates natural, multi-directional cell-cell communication and adhesion. These complex interactions mediate signaling pathways that govern survival, proliferation, and drug efflux, often introducing mechanical cues and resulting in enhanced therapeutic resistance. The core of these 3D structures also develops metabolic gradients (oxygen/nutrients), which further contribute to the observed changes in cytokine expression and the upregulation of immune checkpoints like PD-L1. In addition, the culture method of neurospheres significantly impacts on the maintenance and enrichment of CSCs, characterized by self-renewal potential and increased tumorigenicity *in vivo*. The observed variations suggest that CPZ may have broad clinical applicability, as diverse GBM cell populations (stem cells, bulk cells, varying metabolic phenotypes) could still respond to treatment, overcoming the challenge of GBM’s known inter- and intra-tumor heterogeneity.

In conclusion, our study reveals a previously unrecognized anti-cancer activity of the multifunctional drug CPZ, demonstrating its capacity to enhance the anti-tumor immune response and overcome TMZ resistance in GBM cells. Although our work lacks *in vivo* validation, these powerful preclinical insights directly support the success of our recent Phase II clinical trial (52), in which adding CPZ to the standard first-line regimen led to a clinically meaningful increase in progression-free survival for GBM patients with an unmethylated MGMT promoter.

## Data Availability

The datasets presented in this study can be found in online repositories. The names of the repository/repositories and accession number(s) can be found in the article/**Supplementary Material**.
