# Supplementary material for: Chlorpromazine activates cGAS-STING signaling and reprograms the immune response in glioblastoma
Source: Front Immunol. 2026 Feb 17;17:1743232. doi: 10.3389/fimmu.2026.1743232 (PMC12953537; doi:10.3389/fimmu.2026.1743232)
Supplement: Supplementary file 1 [file Image1.pdf]

A

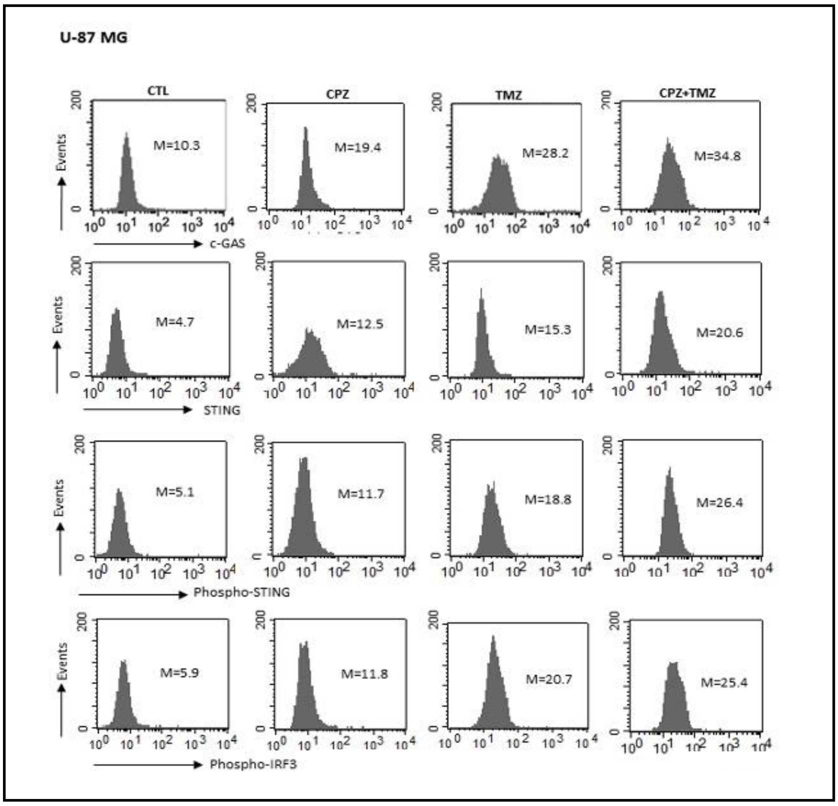

B

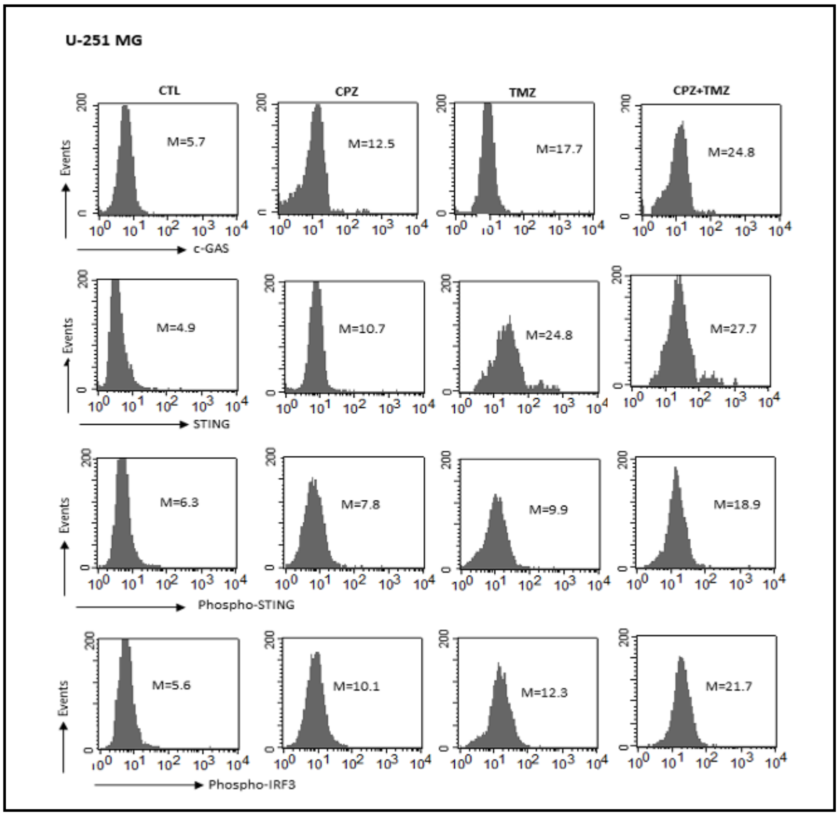

Figure S1 A-B. CPZ and TMZ activate the cGAS-STING signaling pathway in GBM cells. Flow cytometry analyses of the expression of key markers of the cGAS-STING pathway, as indicated. First row, c-GAS; second row, STING; third row, phospho-STING, fourth row, phospho-IRF3. The numbers represent the median fluorescence intensity values obtained in a representative experiment performed in the anchorage-dependent glioblastoma cell lines, U-87 MG in (**A**) and U-251 MG in (**B**), respectively.

C

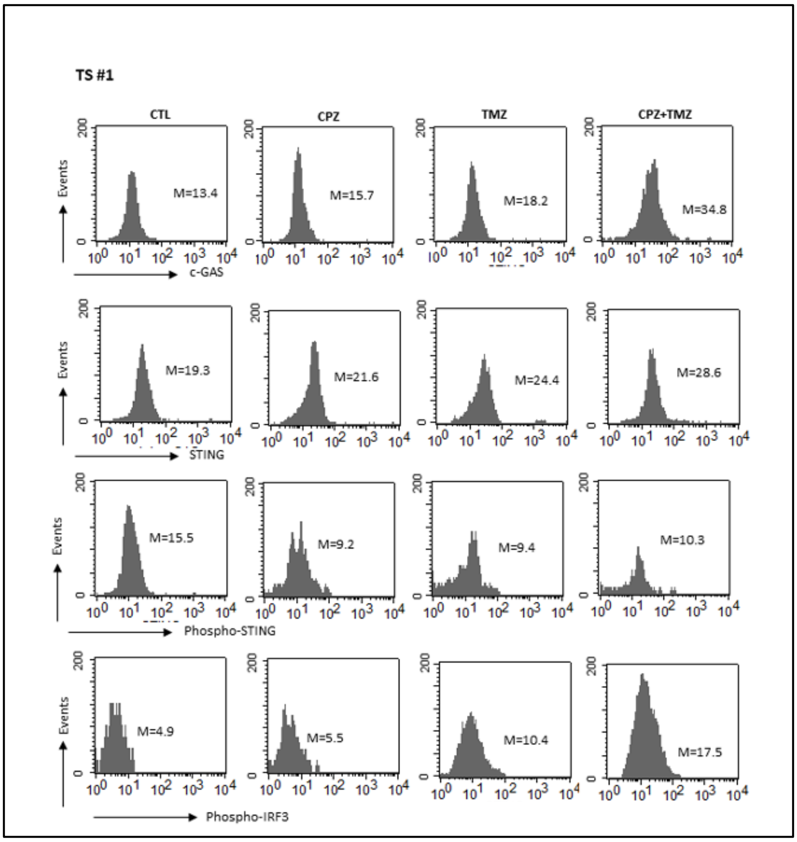

D

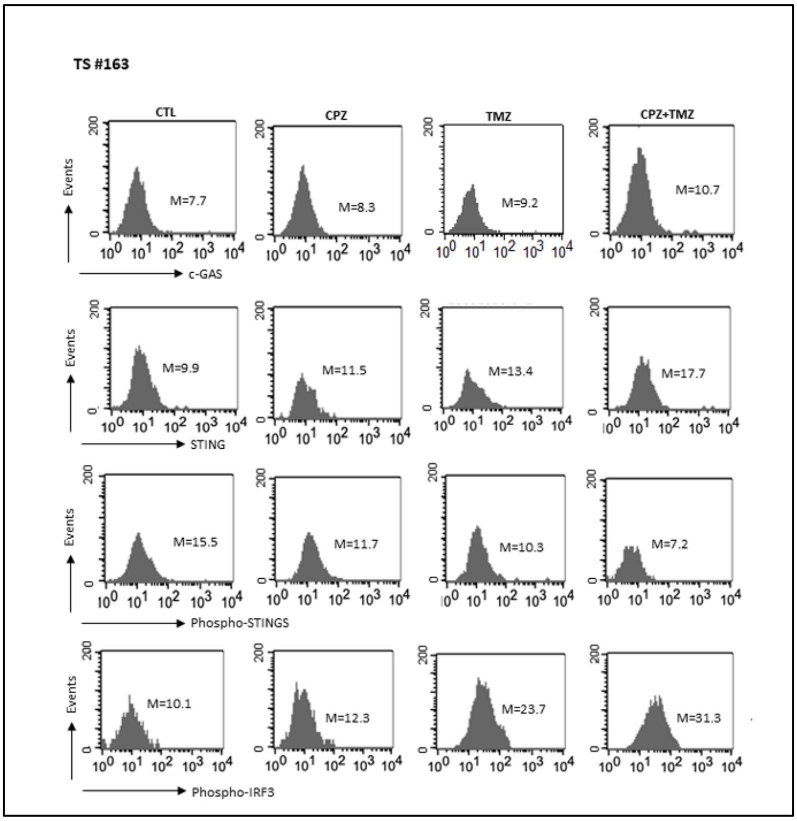

Figure S1 C-D. CPZ and TMZ activate the cGAS-STING signaling pathway in GBM cells. Flow cytometry analyses of the expression of key markers of the cGAS-STING pathway, as indicated. First row, c-GAS; second row, STING; third row, phospho-STING, fourth row, phospho-IRF3. The numbers represent the median fluorescence intensity values obtained in a representative experiment performed in the patient-derived neurospheres, TS #1 in (**C**) and TS #163 in (**D**), respectively.

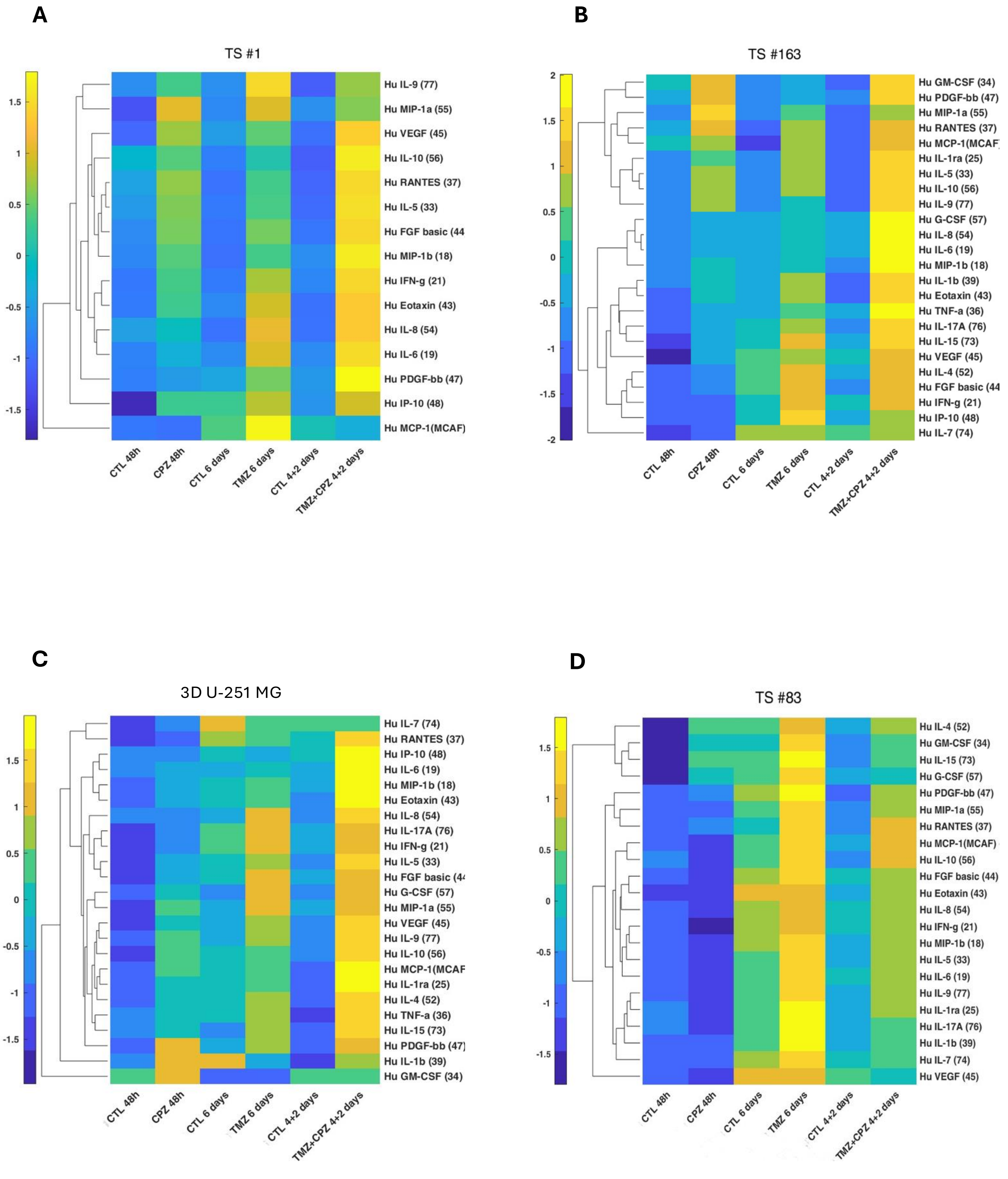

Figure S2. Drug-mediated alterations of GBM spheroids cytokinome. Supervised hierarchical clustering of cytokine levels in conditioned media from patient-derived GBM neurospheres. Cells were treated with CPZ for 48h, TMZ for 6 days, or a combination of TMZ and CPZ (4+2 days). Cytokine luminescence intensity was standardized, normalized to cell counts, and clustered using Euclidean distance.

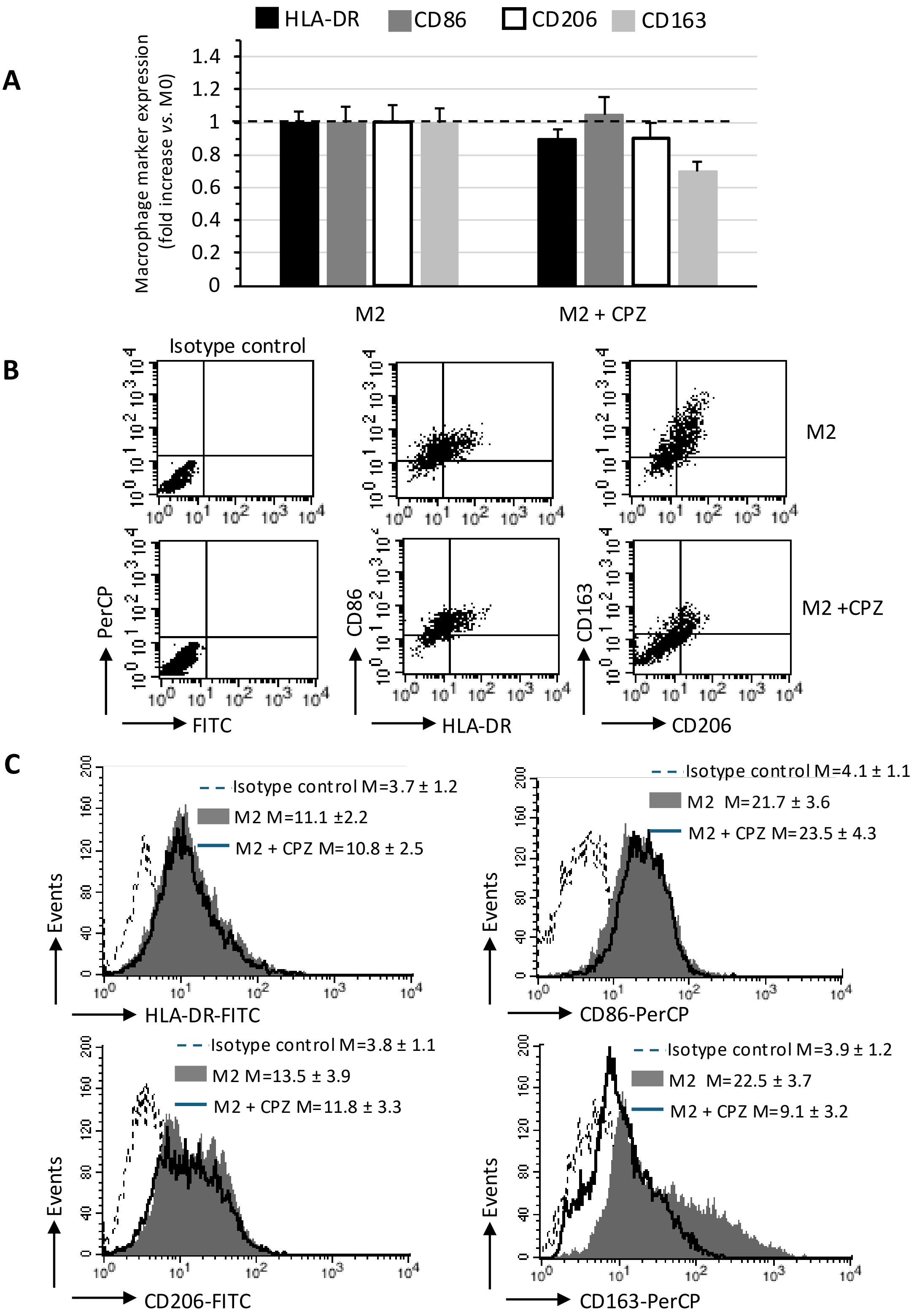

Figure S3. CPZ inhibits macrophage differentiation toward the M2-like phenotype induced by TMZ. Flow cytofluorimetry analyses of plasma membrane expression of HLA-DR, CD86, CD206 and CD163 in living macrophages. (**A**) The expression of the markers is reported in relation to M2 macrophages, in which the median fluorescence intensity values obtained were set equal to 1. (**B**) Dot plots from a representative experiment following double labeling of untreated M2 macrophages (upper row) or treated with CPZ (bottom row). The first column shows isotype controls, the second column shows cells double labeled with anti-HLA-DR and anti-CD86, and the third column shows cells labeled with anti-CD206 and anti-CD163, as indicated. In the upper right quadrant, double positive HLA-DR/CD86 or CD206/CD163 cells are enclosed. (**C**) The histograms show the expression of the four markers separately in the different experimental conditions. The numbers represent the average of the median fluorescence intensity values obtained in three independent experiments ± SD.

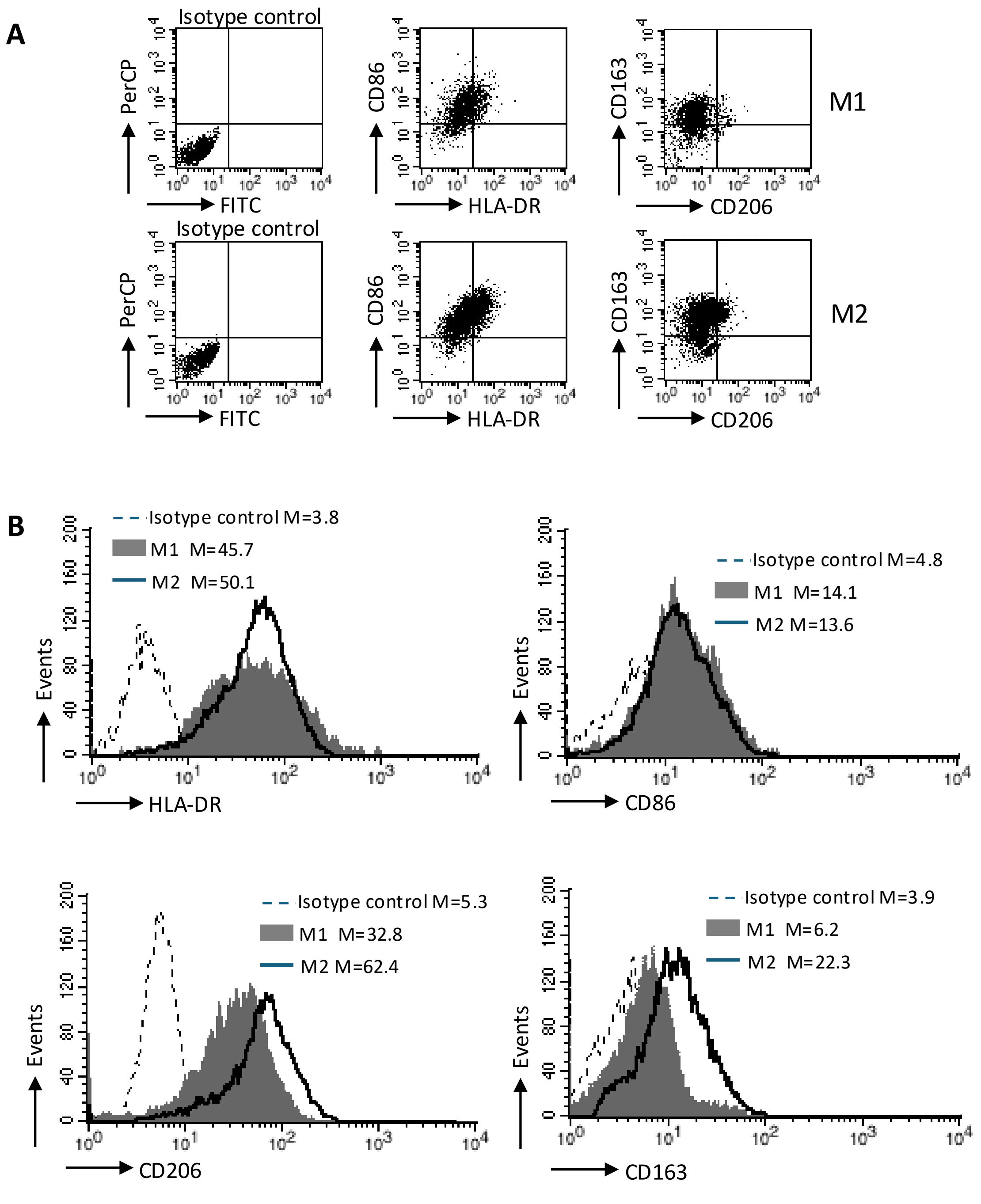

Figure S4. Phenotypic analysis of M1 and M2 macrophages. Flow cytometric analysis of the plasma membrane expression level of HLA-DR, CD86, CD206, and CD163 in M1 and M2 macrophages. (**A**) Dot plots of a representative experiment after double-staining of cells with anti-HLA-DR and anti-CD8 (middle column) or with anti-CD206 and anti-CD163 (right column), as indicated. Negative controls (isotype control) are shown in the left column. (**B**) Histograms showing the expression of the four markers separately in M1 (solid gray curves) and M2 (black curves) macrophages, as specified in the legend. Numbers represent the median fluorescence intensity values obtained in macrophages from a representative healthy donor.

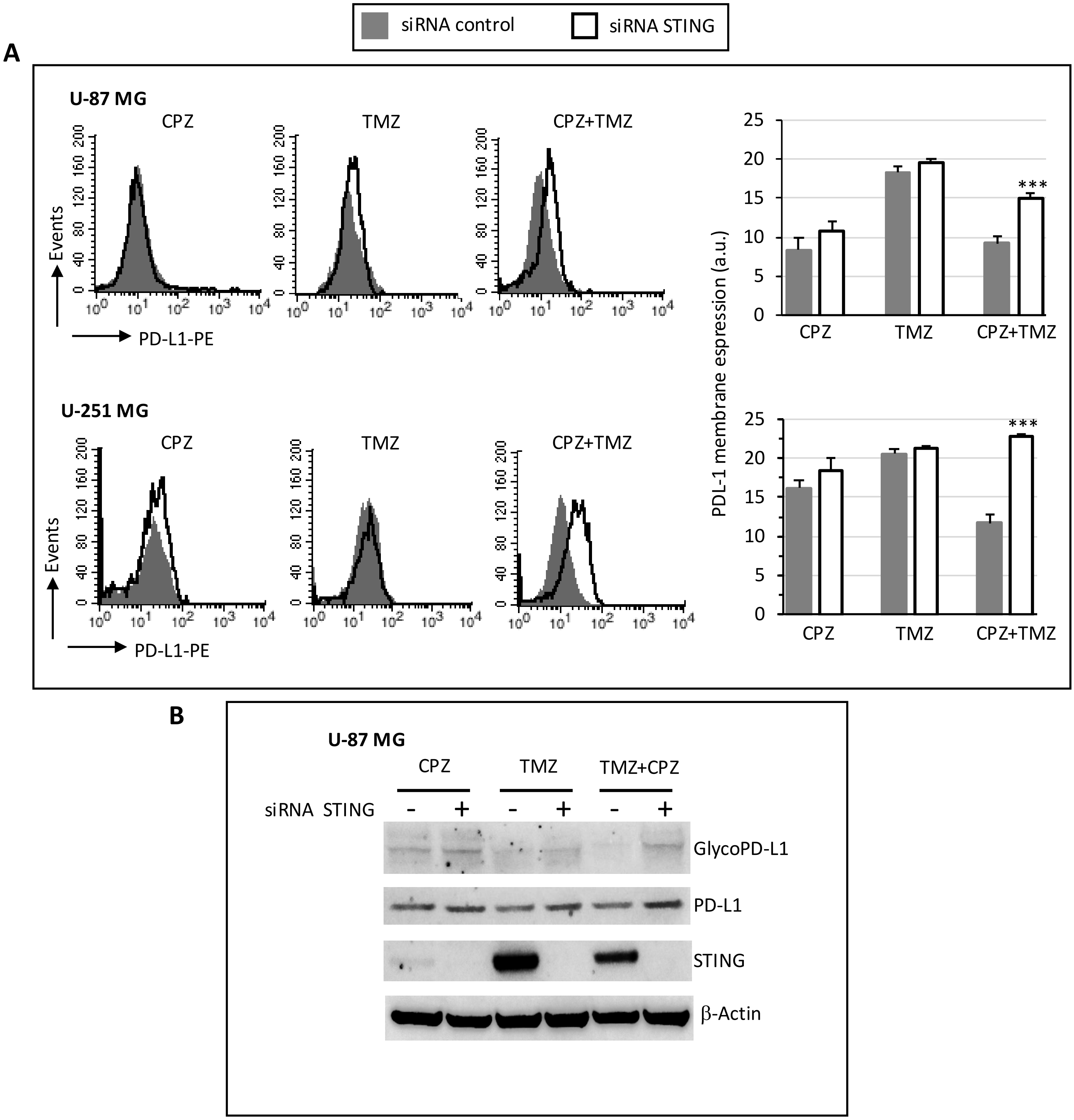

Figure S5. Effect of STING knockdown on PD-L1 expression. (**A**) Flow cytometric analysis of the plasma membrane expression of PD-L1 in U-87 MG (upper panels) and U-251 MG (bottom panels) after transfection with siRNA control (gray solid curves) or siRNA STING (black empty curves) obtained in a representative experiment. Bar graphs show data obtained from three independent measurements and reported as the average of the median fluorescence intensity values ± SD. (***) p<0.001 *vs.* siRNA control as determined by unpaired Student's t-test. (**B**) Representative western blots showing the amount of total and glycosylated isoforms of PD-L1, and STING. β-actin served as the loading control. The protein levels of PD-L1 and STING were assessed in U-87 MG cell line transfected with siRNA control or siRNA STING, as indicated. After transfection, cells were treated with drugs (CPZ for 48 h, TMZ for 6 days, TMZ+CPZ for 4+2 days with IC30 doses), as specified in the Materials and Methods section.
