# Supplementary material for: Chlorpromazine activates cGAS-STING signaling and reprograms the immune response in glioblastoma
Source: Front Immunol. 2026 Feb 17;17:1743232. doi: 10.3389/fimmu.2026.1743232 (PMC12953537; doi:10.3389/fimmu.2026.1743232)
Supplement: Supplementary file 2 [file Table1.docx]

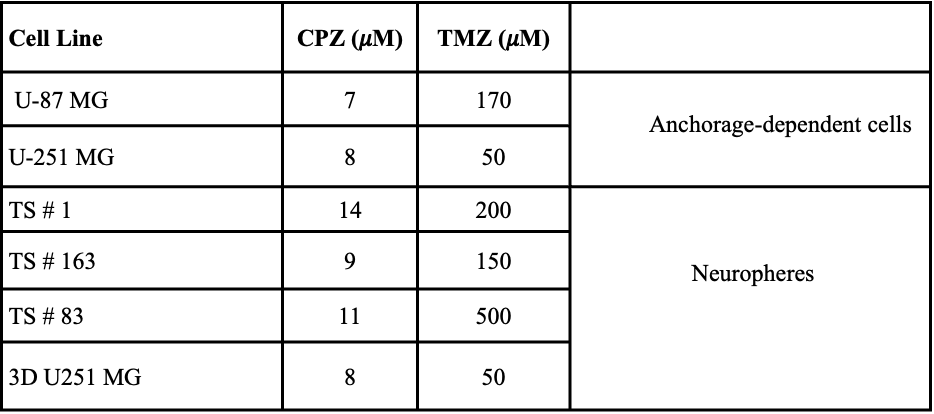


**Supplementary Table 1.** IC30 values for chlorpromazine (CPZ) and temozolomide (TMZ). Table showing μM CPZ and TMZ concentrations corresponding to the IC30 calculated for each cell line.
