# Supplementary material for: Chlorpromazine activates cGAS-STING signaling and reprograms the immune response in glioblastoma
Source: Front Immunol. 2026 Feb 17;17:1743232. doi: 10.3389/fimmu.2026.1743232 (PMC12953537; doi:10.3389/fimmu.2026.1743232)
Supplement: Supplementary file 3 [file Table2.docx]

| **Primers** | **Sequence** |
| --- | --- |
| IFNA1 - Forward | 5'-TCGCCCTTTGCTTTACTGAT-3' |
| IFNA1 - Reverse | 5'-GGGTCTCAGGGAGATCACAG-3' |
| IFNB1 - Forward | 5'-TCCACTACAGCTCTTTCCATGA-3' |
| IFNB1 - Reverse | 5'-TCAAAGTTCATCCTGTCCTTGA-3' |
| IL-6 - Forward | 5'-CGGGAACGAAAGAGAAGCTCTA-3' |
| IL-6 - Reverse | 5'-GGCGCTTGTGGAGAAGGAG-3' |
| IL-12 - Forward | 5'-TGCCTTCACCACTCCCAAAACC-3' |
| IL-12 - Reverse | 5'-CAATCTCTTCAGAAGTGCAAGGG-3' |
| IRF3 - Forward | 5'-AGCAGAGGACCGGAGCAA-3' |
| IRF3 - Reverse | 5'-AGAGGTGTCTGGCTGGGAAA-3' |

**Supplementary Table 2. List of primers.** Table showing the sequence of oligonucleotides used for Real-Time PCR.
